# Evaluating alternative systems of peer review: a large-scale agent-based modelling approach to scientific publication

**DOI:** 10.1007/s11192-017-2375-1

**Published:** 2017-04-03

**Authors:** Michail Kovanis, Ludovic Trinquart, Philippe Ravaud, Raphaël Porcher

**Affiliations:** 1 INSERM U1153, 1 place du parvis Notre Dame, 75004 Paris, France; 2 Université Paris Descartes – Sorbonne Paris cité, Paris, France; 3 Assistance Publique-Hôpitaux de Paris, Hôpital Hôtel-Dieu, Centre d’Epidémiologie Clinique, Paris, France; 4 Cochrane France, Paris, France; 5 Department of Epidemiology, Columbia University Mailman School of Public Health, New York, USA

**Keywords:** Peer review, Cascade, Portable, Post-publication, Complex systems, Agent-based model

## Abstract

The debate on whether the peer-review system is in crisis has been heated recently. A variety of alternative systems have been proposed to improve the system and make it sustainable. However, we lack sufficient evidence and data related to these issues. Here we used a previously developed agent-based model of the scientific publication and peer-review system calibrated with empirical data to compare the efficiency of five alternative peer-review systems with the conventional system. We modelled two systems of immediate publication, with and without online reviews (crowdsourcing), a system with only one round of reviews and revisions allowed (re-review opt-out) and two review-sharing systems in which rejected manuscripts are resubmitted along with their past reviews to any other journal (portable) or to only those of the same publisher but of lower impact factor (cascade). The review-sharing systems outperformed or matched the performance of the conventional one in all peer-review efficiency, reviewer effort and scientific dissemination metrics we used. The systems especially showed a large decrease in total time of the peer-review process and total time devoted by reviewers to complete all reports in a year. The two systems with immediate publication released more scientific information than the conventional one but provided almost no other benefit. Re-review opt-out decreased the time reviewers devoted to peer review but had lower performance on screening papers that should not be published and relative increase in intrinsic quality of papers due to peer review than the conventional system. Sensitivity analyses showed consistent findings to those from our main simulations. We recommend prioritizing a system of review-sharing to create a sustainable scientific publication and peer-review system.

## Introduction

The peer-review system is undeniably the gold standard of scientific publication. It serves a double purpose; to screen out bad science and to improve the quality of manuscripts before they are published. However, the scientific community is concerned about the sustainability of the system given the growing number of papers submitted for publication, which puts pressure on the system (Bohannon 2013; Hopewell et al. 2014; Arns 2014; Jennings 2006; Mulligan et al. 2013; Nicholas et al. 2015; Rennie 2016; Sense About Science 2012; Siler et al. 2015; Walker and Rocha da Silva 2015b; Kovanis et al. 2016b).

Much effort has been devoted to proposing alternative systems of peer review or interventions to improve it. However, little effort has focused on testing or evaluating the effectiveness of the alternative systems. Currently, BMC Biology has implemented re-review opt-out, whereby authors are allowed to opt out from a second round of peer review after major revisions to their paper. The journal of Atmospheric Chemistry and Physics has implemented immediate publication upon submission of an article, with online and invited reviews. Philica and F1000 research are also implementing a similar model. Pre-publication servers such as ArXiV or bioRxiv allow researchers to upload their papers before submitting them to a peer-reviewed journal. The Nature and JAMA groups give scientists the option to allow editors of journals within each respective group to discuss rejected manuscripts and to propose submission to another journal of the group (Walker and Rocha da Silva 2015b; Cals et al. 2013; Gura 2002; Houry et al. 2012; Patel 2014; Stahel and Moore 2014; van Rooyen et al. 1999; Ware 2013).

Until 2016, only 22 randomized controlled trials had been conducted to assess peer-review interventions such as double-blind peer review and the addition of a statistical reviewer (Bruce et al. 2016). Studying all the proposed and already-implemented alternatives is not easy. Putting all of them under a real-life test would be costly, time-consuming and sometimes not feasible. Thus, we need approaches such as computer simulations that would allow for quicker screening to identify the most promising alternatives to the peer-review system to be later examined in a real-life test.

Because of the highly complex nature of the scientific publication system, here we used techniques from complex systems modelling, specifically agent-based modelling (ABM), to describe the system. Because of multiple interactions of many heterogeneous and independent agents (e.g., authors, editors, reviewers, papers), this sort of systemic thinking and detailed microscopic modelling was necessary (Galea et al. 2010; Vespignani 2012; Bonabeau 2002; Marshall and Galea 2015). Author, editor and referee behaviour has been extensively studied with ABM and other modelling approaches. Some authors focused on how the number of reviewers, reciprocity, rationality and other motives between referees and authors affect the quality of peer review, and others redesigned models to replicate their results (Bianchi and Squazzoni 2015; Squazzoni and Gandelli 2013; Thurner and Hanel 2011; Paolucci and Grimaldo 2014; Righi and Takács 2017). Others modelled how objectivity and subjectivity in reviewers’ decisions macroscopically bias peer review (Park et al. 2014) or estimated the level of bias necessary to affect peer review in grant applications (Day 2015). There have also been attempts to model alternative peer-review systems in a one-journal or systemic approach (Herron 2012; Allesina 2012). Most of these works have focused on specific questions about peer review, often reviewer behaviour, without considering the complete scientific publication system and without calibration with empirical data. However, to improve the peer-review system, we need to adopt a unified approach to both scientific publication and peer review that is more holistic and to use empirical data for calibrating models. Therefore, we have developed an ABM that we calibrated with empirical data pertaining to the biomedical domain (Kovanis et al. 2016a).

Here, our objective was to use an agent-based model to evaluate the efficiency of alternative peer-review systems currently implemented by some biomedical and general journals. We modified the ABM we previously developed to match the behaviour of these alternatives and compared their performance in terms of the base model. To our best knowledge, previous models focused mostly on microscopic behaviours; here we selected widely discussed systems requiring more macroscopic modifications to the ABM, which are largely understudied. Section ("Methods") contains a brief description of the base model for the conventional system, the alternative peer-review systems, their real-life examples and the changes we implemented in the sub-models of the conventional system. In  “Results” section we present our results and our exploration of the parameter space. Finally, in “Discussion” section we discuss the implications of our results.

## Methods

### Overview

We used a previously developed ABM that was calibrated with empirical data and adopts a unified approach of scientific publication and peer review (Kovanis et al. 2016a). This ABM was structured in independently parameterized sub-models pertaining to the submission and peer-review process. Structural changes to some of these sub-models allowed us to model the alternative peer-review systems.

**Fig. 1:**
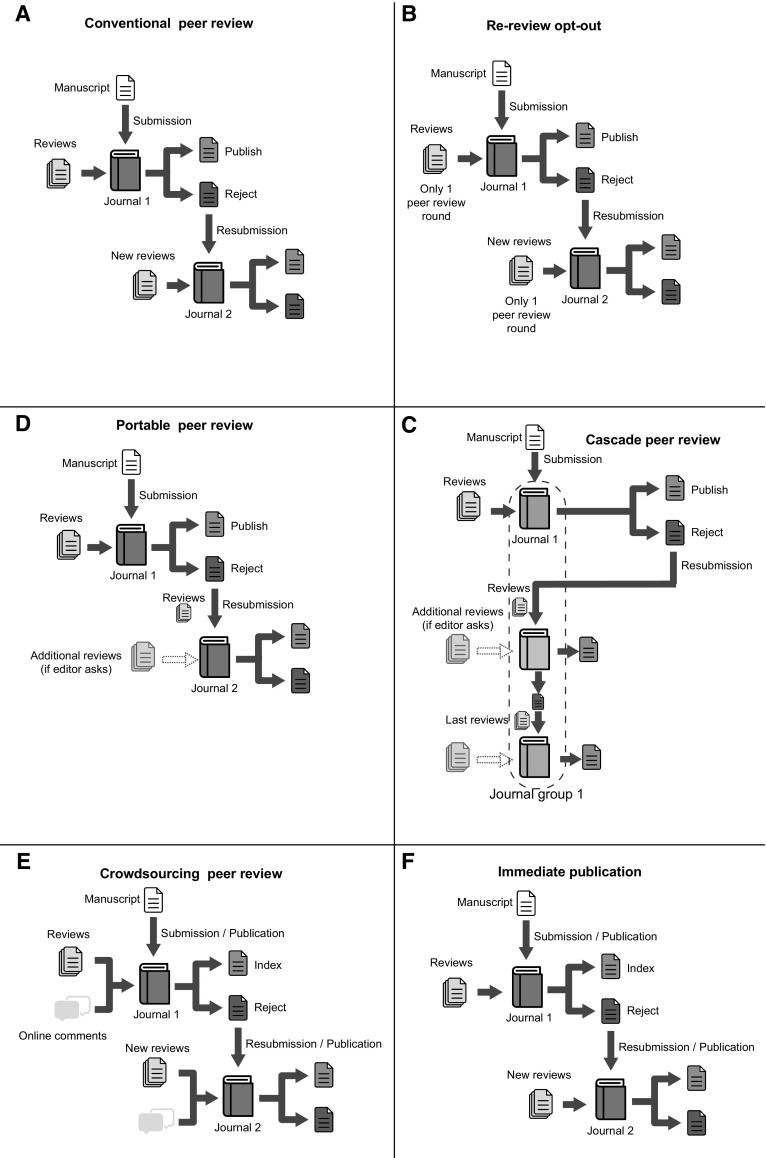
Diagrams of the alternative peer-review systems

We compared five alternative systems of peer review discussed in the literature and to some extent already implemented by some journals and publishers: re-review opt-out, cascade peer review, portable peer review, crowdsourcing peer review, and immediate publication (Fig. 1). Their main characteristics and parameters are summarized in the Table 1.

**Table 1:** Summary of the characteristics and parameters of the alternative peer-review systems

Peer-review systems	Main characteristics	Differences from the conventional system
Re-review opt-out	Only one round of peer review and revisions Acceptance or rejection depends on editor’s evaluation of the revisions	*Evaluation of papers* Only 1 one round of peer review and revisions *Acceptance or rejection of papers* If paper is not rejected by the reviewers, then the editor evaluates (*Q*_*e*_) its revised version (*Q*_revised_) $$ Q_{e} \mathop \leftarrow \limits^{{\begin{array}{*{20}c} {\text{Uniformly }} \\ {\text{drawn}} \\ \end{array} }} \left[ {0.9Q_{\text{revised}} ,1.1Q_{\text{revised}} } \right] $$ Accepted only if the editor’s evaluation is higher or equal to the acceptance threshold (*T*_max_) of the journal (*j*)
Cascade	Sharing of past reviews between journals belonging to the same group Resubmissions are allowed only in journals of the same publisher and of lower reputation	*Journals* Each journal belongs to one of the 4 groups that shares reviews internally *Decision on whether to ask for new reviews or not* The journal receives a paper of scientific value *Q*, its past reviews (*Q*_*r*_) and the editor issues an evaluation (*Q*_*e*_) If *Q* ≥ *T*_max_ the paper is immediately accepted If $\frac{{\left| {Q_{e} - Q_{r} } \right|}}{{Q_{r} }} \le 0.1$ the authors revise the paper and then the editor re-evaluates it and decides on acceptance or not If $\frac{{\left| {Q_{e} - Q_{r} } \right|}}{{Q_{r} }} > 0.1$ the editor asks for new reviews *Resubmission probability* The probability of resubmission (*P*_res_) depends on whether the number of submissions (*N*_sub_) is higher than in the conventional system $P_{\text{res}} = 0.88^{{\left( {N_{\text{sub}} - 1} \right)/2}}$ instead of $P_{\text{res}} = 0.88^{{\left( {N_{\text{sub}} - 1} \right)}}$ *Journal to resubmit* Randomly selected among the next 5 journals of lower reputation (belonging to the same group)
Portable	Sharing of past reviews between journals	*Decision on whether to ask for new reviews or not* Same as in the cascade system *Resubmission probability* Same as in the cascade system
Crowdsourcing	Publication as “discussion papers” upon submission Editor takes into account possible online comments	*Initial scientific information (* ${\text{SI}}_{\text{init}} = {\text{AR}}_{j} \times Q$ *)* New submissions release initial scientific information depending on their scientific value (*Q*) and the journal (AR_*j*_) *Evaluation of papers* Papers are evaluated by invited reviewers (*N*_*R*_) and by a certain number of online commenters equal to $\frac{{{\text{SI}}_{\text{init}} }}{{{\text{mean}}\left( {{\text{SI}}_{\text{total}} } \right)^{2} }}$ mean (SI_total_) is the average initial scientific information for all submissions in a time step *Acceptance or rejection of papers:* The editor evaluates the papers using the mean evaluation value of all the online comments (*Q*_online_) and the evaluation of the invited reviewers (*Q*_invited_) $Q_{r} = \frac{{Q_{\text{online}} + N_{R} Q_{\text{invited}} }}{{N_{R} + 1}}$ If the paper receives no online comments, then *Q*_*r*_ = *Q*_invited_ *Final scientific information (* ${\text{SI}} = {\text{IF}}_{j} \times Q_{F}$ *)* All published papers release the rest of their scientific information (SI − SI_init_) at the time of acceptance For papers rejected and not resubmitted, 80% of their SI_init_ is removed from the system
Immediate publication	Publication as “discussion papers” upon submission	*Initial scientific information* Same as in the crowdsourcing system *Final scientific information* Same as in the crowdsourcing system

### Model for the conventional publication and peer-review system

Here we provide a brief description of our ABM of the conventional scientific publication and peer-review system. For a more detailed description, see Kovanis et al. (2016a).

We characterized *N* researchers by resources *R*(*t*) and scientific level *S*(*t*). The scientific level was defined as *S*(*t*) = *R*(*t*) + *S*_*b*_(*t*), where *t* the time step and *S*_*b*_(*t*) the sum of all the rewards that a researcher can receive to determine scientific level. The resources represent all the means that researchers have at their disposal for conducting research. The scientific level expresses a researcher’s experience and capacity to conduct better research.

Manuscripts were characterized by an intrinsic quality score (*Q* score), which serves as a proxy for their intrinsic scientific value but also their disruptive, innovative, or controversial nature as well as quality of reporting. At each time step, *N*_*s*_ randomly selected researchers submitted their paper. At the time of submission (*t*_*s*_) of their paper, authors would lose an amount of resources *R*_inv_ associated with the conduct of the research reported in that paper with $0.2R\left( {t_{s} } \right) \le R_{\text{inv}} \le 0.7R \left( {t_{s} } \right)$. Each paper had an initial expected quality *E*_*Q*_ defined as:

$$ E_{Q} = 0.8\frac{{0.1R_{\text{inv}} }}{{0.1R_{\text{inv}} + 1}} + 0.2\frac{{0.01S\left( {t_{s} } \right)}}{{0.01S\left( {t_{s} } \right) + 1}} $$

The weights were chosen to represent the greater contribution of invested resources to the scientific level and to not allow the magnitude of $S\left( {t_{s} } \right)$ to surpass the final $E_{Q}$ value. The *Q* score was drawn from a normal distribution $Q\,\sim\,N \left( {E_{Q} ,\; 0.1\;E_{Q} } \right)$. This score determines how a researcher chooses a target journal and drives in-house and external peer-review assessments.

We characterized *J* journals by 3 state variables: a reputation value (we used rescaled impact factors) and by related rejection or acceptance thresholds, $T_{\hbox{min} }^{j} < T_{\hbox{max} }^{j} , j = 1, \ldots , J$. We assumed that authors had a general knowledge of journal standards and, given the *Q* score, would try to obtain the most recognition from their work. Hence, the journal for the first submission was chosen at random among those with *T*_*min*_^*j*^ within the asymmetrical range $Q - 0.45 \varepsilon \le T_{\hbox{min} }^{j} \le Q + 0.55\varepsilon$, where $\varepsilon \sim2 \times N \left( {\frac{Q}{5},\frac{Q}{20}} \right)$. This process resulted in a slight trend of high targeting in every first submission.

We drew the editor’s assessment of the manuscript *Q*_*e*_ from a uniform distribution over $\left[ {0.9Q; \;1.1Q} \right]$. If *Q*_*e*_ < *T*_min_^*j*^, the manuscript could be rejected without external peer review. If *Q*_*e*_ ≥ *T*_min_^*j*^, the manuscript was sent for external peer review to 2 or 3 reviewers. The reviewers’ assessments were defined as $Q_{r} \,\sim\,N \left( {Q - c,\;r \times Q} \right)$, where *r* was a random error and *c* measured the competitiveness of the reviewer. We defined *r* = *r*_*r*_ + *r*_*j*_ − *r*_*Q*_, where *r*_*r*_ is the reviewing error, *r*_*j*_ the journal error and *r*_*Q*_ the score error. With 65% probability, we set *r*_*t*_ = 0.1; with 12%, *r*_*t*_ = 0.05; and with 13%, *r*_*t*_ = 0.01. We drew *r*_*j*_ randomly from a uniform distribution over [0; 0.15], where *r*_*j*_ = 0 corresponded to the highest reputation journal and *r*_*j*_ = 0.15 to the lowest. Finally, *r*_*Q*_ = 0.05 × *Q*. We assumed that a competitive behavior would occur more often for journals with higher reputation. The probability of appearance ranged uniformly from 10 to 66%, where *c* was drawn randomly from a uniform distribution over [0.01; 0.05].

We randomly selected one of the reviewers’ evaluations as a proxy of the editor’s opinion. If *Q*_*r*_ ≥ *T*_max_, the manuscript was accepted and if *Q*_*r*_ ≤ *T*_min_, it was rejected. When *T*_min_ ≤ *Q*_*r*_ < *T*_max_, the author was asked to revise the manuscript before a second round of peer review. In the latter case, the author invested an extra amount of resources $R_{\text{imp}} \,\sim\,N\left( {\frac{8}{60},\frac{1}{60}} \right) \times \left( {R - R_{\text{inv}} } \right)$. The cumulative amount of invested resources was used to derive a new *Q* score as before. The manuscript was re-evaluated and accepted only if $Q_{r} \ge T_{\hbox{max} }$. The probability of resubmission *P*_res_ after a rejection decreased with increasing number of resubmissions *r*, *P*_res_ = 0.88^*r*−1^. After the first rejection, the authors would target journals of lower reputation. Thus, they randomly selected journals in the (symmetrical this time) range $0.22Q - 0.5\varepsilon \le T_{\hbox{min} }^{j} \le 0.22Q + 0.5\varepsilon$, where *Q* is the initial score of the manuscript.

Resources and scientific level were updated at each time step. If an article is published, the author received a random reward $p \times \left( {R_{\text{inv}} + \mathop \sum \limits_{i} R_{\text{imp}}^{i} } \right),\quad  0\,\le\,p\,\le 0.5$, otherwise, the author would permanently lose all the resources invested. In case of publication, the author also received a reward for resources in scientific level. The scientific level of a reviewer was credited with a random reward between 0 and 0.001 every time the reviewer completed a review because of knowledge acquired from the paper. Moreover, at the end of each week, the researchers received an update to their resources and scientific levels randomly drawn between 0.1 and 1, which reflected an increase of the means to conduct research with time.

We assumed that when a paper was published, it released scientific information to the community ${\text{SI}} = {\text{IF}}_{j} \times Q_{F}$, where IF_*j*_ is the reputation value of the journal (*j*) that published it and *Q*_*F*_ is the *Q* score of the paper, after all revisions. Scientific information is a comparative variable and its purpose is to assess the effectiveness of a system in producing more papers of higher *Q* score and in disseminating them to the rest of the scientific community. The reputation value (IF) of a scientific journal is a proxy of the size of the community that will read the paper and the *Q* score a proxy of how much people who read the paper will benefit from it.

### Re-review opt-out

The intent of this system, currently implemented by BMC Biology, is to shorten the time of peer review by allowing authors to opt out from a second round of reviews. Thus, authors with a paper judged publishable with major revisions by the reviewers can choose whether they want their manuscript to be evaluated by the editor only or again by the reviewers after revising it (Robertson 2013).

We chose to model a maximum implementation of this intervention so that authors would always choose to opt out from a second round and therefore all decisions for every submission would be made after at most one peer-review round. For papers undergoing peer review, the authors always revised, and then the editor made an assessment (*Q*_*e*_) of the revised manuscript from a uniform distribution between 0.9 $Q_{\text{revised}}$ and $1.1\;Q_{\text{revised}}$. With *Q*_*e*_ ≥ *T*_max_^*j*^, a paper was accepted; otherwise it was rejected. All other processes were handled as in the conventional system.

### Cascade peer review

When papers are rejected, their authors usually revise them and resubmit to other journals for publishing. In the conventional peer-review system, this implies that the same manuscripts will be reviewed multiple times and their publication can be seriously delayed. To avoid this situation, some publishers have decided to share reviews for rejected manuscripts among the journals they manage, thus avoiding redundant reviews and shortening the evaluation time. Such publishers include Nature Publishing Group, JAMA, BioMed Central and British Medical Journal (Walker and Rocha da Silva 2015a; Cals et al. 2013; Van Noorden 2013).

We randomly allocated 105 journals of various reputation value to one of four arbitrary publisher groups. We assumed that every journal belonged to one of these groups. Each journal was allocated to one of the publisher groups by using a categorical distribution with parameters (probability of belonging to each group) drawn from a normal distribution $\sim N\left( {0.25,0.025} \right)$ for the three first groups, with the remaining ones allocated to the fourth.

When a paper was rejected, the editor proposed that the author send it to journals of the same network but of lower reputation. We assumed that if authors decided to resubmit, then they never rejected this proposal. Then, one of the next five journals of lower reputation value (of the same network) was randomly selected and the manuscript was resubmitted to it, along with the last evaluation value (*Q*_*r*_).

The new editor immediately accepted the resubmitted paper without asking for further reviews if $Q_{r} \ge T_{\hbox{max} }$; otherwise, the editor asked for revisions if $\frac{{\left| {Q_{e} - Q_{r} } \right|}}{{Q_{r} }} \le 0.1$, where *Q*_*e*_ is the editor’s assessment of the manuscript (drawn uniformly from between 0.9*Q* and 1.1*Q*). Then the editor re-assessed the paper and decided whether to accept or reject it. Papers rejected were more likely to be resubmitted in this system than in the conventional system; thus the probability of resubmission was modified as $P_{\text{res}} = 0.88^{{\left( {N_{\text{sub}} - 1} \right)/2}}$. Authors cascaded their submissions always using the last reviews they obtained. With $\frac{{\left| {Q_{e} - Q_{r} } \right|}}{{Q_{r} }} > 0.1$, the editor asked for new reviews and the submission was handled as in the base model.

### Portable peer review

In this system, the authors resubmit their rejected manuscripts along with the reviews they received from their last peer-reviewed submission (if any). In contrast to the cascade system, the journals were not organized in groups and thus the authors sent their previous reviews to any of the journals they would be resubmitting to as in the conventional system. Based on the same rule as in the cascade system, editors could choose to ask for new reviews or revisions before deciding on acceptance or rejection.

### Crowdsourcing peer review (Immediate publication with online and invited reviews)

Crowdsourcing online reviews is implemented in part by various journals such as F1000Research, Philica and the Semantic Web Journal. The purpose of this system is the immediate release of scientific information and the more accurate evaluation of papers because of any additional online comments or reviews. The journal of Atmospheric Chemistry and Physics (ACP) is also a well-known example of the use of such a system. Papers submitted to ACP pass a quick editorial pre-screening and are almost immediately published, following their submission, in the journal’s website as “discussion papers”. A published paper is then assigned external peer reviewers. The peer reviewers start an online discussion with the authors and other interested members of the scientific community. After a fixed number of weeks, the discussion stops and the authors revise the paper and resubmit it for publication (Walker and Rocha da Silva 2015a; Pöschl 2012; Journal 2015; Hunter 2012).

In our approach, papers were subject to traditional editorial assessment instead of a quick editorial pre-screening. This discussion did not have any pre-specified time limit and the rejected manuscripts could be left on the journal’s webpage or resubmitted to another journal.

Each manuscript that passed the conventional in-house review stage was immediately published, along with a call for online reviews/comments and the traditional invitation to two or three external reviewers selected by the journal’s editor. Every fresh submission released an initial amount of scientific information, ${\text{SI}}_{\text{init}} = {\text{AR}}_{j} \times {\text{Q}}$, where ${\text{AR}}_{j}$ represents the reputation of the “discussion papers” section of the journal (*j*). We obtained ${\text{AR}}_{j}$ from the original simulations of the conventional system, and it is equal to the acceptance rate of papers, after the editorial screening process. We assumed that a publication attracted a number of online reviewers equal to $\frac{{{\text{SI}}_{\text{init}} }}{{{\text{mean}}\left( {{\text{SI}}_{\text{total}} } \right)^{2} }}$, rounded to the nearest integer, where ${\text{mean}}\left( {{\text{SI}}_{\text{total}} } \right)$ is the average ${\text{SI}}_{\text{init}}$ of all papers submitted at each time step (${\text{SI}}_{\text{total}}$ represents the distribution of ${\text{SI}}_{\text{init}}$ values at a time step).

The online commenters evaluated the paper in the same way as the normal reviewers. The editor averaged the scores of the online commenters ($Q_{\text{online}}$) and randomly selected one of the invited reviewers’ scores ($Q_{\text{invited}}$), as in the conventional system to make a decision ($Q_{\text{r}}$). We assumed that editors took more into account comments from reviewers they invited than uninvited reviewers, thus $Q_{r} = \frac{{Q_{\text{online}} + nQ_{\text{invited}} }}{{n{ + 1}}}$, where n is the number of invited reviewers. Thus, the more online reviewers, the greater the chance a paper was more accurately evaluated. If the paper did not attract any online comments, then $Q_{r} = Q_{\text{invited}}$.

With $Q_{r} \ge T_{\hbox{max} }$, the paper was revised once, considered indexed in the bibliographical databases (Web of science, MEDLINE etc.) and included as a part of the next issue of the journal, thus releasing the rest of its scientific information at the time of indexation. With $Q_{r} < T_{\hbox{max} }$, the authors decided to resubmit based on $P_{\text{res}} = 0.88^{{N_{\text{sub}} - 1}}$ or leave their paper unindexed on the webpage of the journal. In the latter case, the paper would still release some scientific information because it can be found online but less so because it will be hidden in the journal’s website. Thus, subtracting an amount from the total scientific information the scientific community had already accumulated (because of the paper’s higher visibility as a “discussion” paper), the manuscript’s final scientific information becomes ${\text{SI}} = 0.2\;{\text{SI}}_{\text{init}}$.

### Immediate publication

In the system of immediate publication, papers are immediately available to the readers as “discussion papers” before they are peer reviewed via the webpage of the journal. This system is similar to the crowdsourcing system (“Crowdsourcing peer review (Immediate publication with online and invited reviews)” section) but without assuming that editors would take into consideration any online reviews or comments.

### Implementation and system comparison

We programmed the models by using MATLAB (MATLAB and Statistics Toolbox Release 2016b, The MathWorks, Inc., Natick, MA, USA) with a total number of researchers *N* = 25,000, total number of journals *J* = 105 and weekly submissions drawn from a normal distribution ~*N* (850, 85) (each simulation week is 1 time-step). We ran the simulations for 10 years, with a burn-in period of 1 year for the initialization of the model. All main results were averaged over 100 simulation runs. Code is available at http://www.clinicalepidemio.fr/peerreview_alternative_systems/.

We defined three different types of outcomes to compare all alternative systems with the conventional system; peer-review efficiency, reviewer effort and scientific dissemination. Peer-review efficiency corresponded to the double purpose of peer review. We measured it by using the separation of the *Q* score distributions of the published and unpublished papers and the relative improvement in average *Q* score for all papers after revision as compared to that for the first submission. We used the Hellinger distance as a quantifying measure of the overlap between two distributions: the higher the Hellinger distance, the less the overlap (Nikulin 2001). We measured reviewer effort by using the total time reviewers devoted to peer review in a year. We obtained this outcome in hours from our simulations and transformed it into working years per year with the following equation:

$$ {\text{time spent in peer review}} = \frac{{{{{\text{hours devoted to}}\;{\text{peer review}}} \mathord{\left/ {\vphantom {{{\text{hours devoted to}}\;{\text{peer review}}} {\text{work hours}}}} \right. \kern-0pt} {\text{work hours}}}}}{{{\text{year}} - {\text{weekends}} - {\text{holidays}}}} $$

where ${\text{work hours}} = 8\,{\text{h per day}}$, ${\text{year}} = 365\,{\text{days}}$, ${\text{weekends}} = 104\,{\text{days}}$ and ${\text{holidays}} = 25.3\,{\text{days}}$ (average paid holidays in 21 OECD countries) (Ray and Schmitt 2007). Finally, we measured scientific dissemination by using the number of annual publications, the median weeks between first submission of a paper and the final decision, the average *Q* score for all papers and the average weekly release of scientific information. For estimating the two time-related measures, we used the respective distributions from an international survey of 4000 participants (Mulligan et al. 2013).

Finally, we considered that a peer-review system was beneficial if it improved any of the outcomes without deteriorating the peer-review efficiency and more efficient than the conventional if it improved all types of outcomes.

### Sensitivity analyses

We performed sensitivity analyses of two of the alternative peer-review systems: cascade and crowdsourcing. We excluded the re-review opt-out, portable and immediate publication systems because the first is already at its maximum configuration and cannot realistically be improved in our ABM and the second and third can be considered special cases of the cascade and crowdsourcing systems, respectively. These analyses focused on identifying the effect of different configurations of the cascade and crowdsourcing systems on their outputs. All sensitivity analyses were averaged over 10 simulation runs.

#### Exploring the parameter space for the cascade peer-review system

In the main version of the cascade system, with initialized *N*_*g*_ = 4 journal groups, the editor asks for new reviews or not based on $\frac{{\left| {Q_{e} - Q_{r} } \right|}}{{Q_{r} }} \le \alpha$, where *α* = 0.1, and the probability that an author accepts the editor’s proposal is *P*_cas_ = 1.0. We explored the parameter space by varying these three parameters one at a time while keeping the other two the same as in the main version of the cascade system. We ran the cascade system for $N_{g} = 2, 3 \;{\text{and}}\; 5$, for *α* = 0.0 and 1.0 and for $P_{\text{cas}} = 0.7, 0.8$ and 0.9. The cases with *α* = 0.0 and 1.0 represent those for which all and none of the resubmitted papers receive new peer review, respectively.

#### Effect of the editor’s decision and online comments with the crowdsourcing system

We explored different assumptions on how editors decide on acceptance or rejection of a paper and how the online comments affect the system overall. Here we explored the cases in which all papers received 1, 5 and 20 comments. Moreover, we simulated when editors averaged all reviews, online and invited, and when they chose at random one of the online or invited peer reviews to represent their decision. The last two cases assumed a mechanism of attracting online comments identical to the main version of the system.

## Results

### Peer-review efficiency (Table 2)

Only the cascade and the crowdsourcing peer-review systems outperformed the conventional system for both outcomes. Their performance was similar in terms of separation of *Q* score distributions; however, the cascade system outperformed both the conventional and crowdsourcing systems in terms of improving the *Q* scores of submitted papers and the average weekly release of scientific information. The immediate publication system performed almost identically to the conventional system, and the portable and re-review opt-out systems failed to match that of the conventional system in one and two of the measures, respectively.

**Table 2:** Values of all outcome measures of all peer-review systems implemented

Outcome measures	Conventional	Re-review opt-out	Cascade	Portable	Crowdsourcing	Immediate publication
*Peer*-*review efficiency*						
Separation of the *Q* score distributions of the published and unpublished papers (HD)	0.433 ± 0.003	0.312 ± 0.105 (−28.0%)	0.452 ± 0.008 (+4.4%)	0.414 ± 0.004 (−4.2%)	0.448 ± 0.003 (+3.5%)	0.447 ± 0.003 (+3.2%)
Relative improvement of *Q* score of papers after revisions	5.51 ± 0.03%	3.32 ± 0.03% (−39.6%)	6.27 ± 0.04% (+13.9%)	5.96 ± 0.02% (+8.1%)	5.66 ± 0.03% (+2.7%)	5.65 ± 0.03% (+2.6%)
*Reviewer effort*						
Time spent in peer review (work years/year)	971 ± 12	684 ± 9 (−20.5%)	360 ± 6 (−62.9%)	347 ± 6 (−64.3%)	992 ± 12 (+2.1%)	995 ± 14 (+15.7%)
*Scientific dissemination*						
Annual no. of publications	31,425 ± 164	33,743 ± 165 (+7.4%)	29,757 ± 389 (−5.3%)	33,614 ± 184 (+7.0%)	31,143 ± 158 (−0.9%)	31,199 ± 178 (−0.7%)
Time between first submission and final decision (weeks), median	15	14 (−6.7%)	7.9 ± 0.2 (−47.3%)	8 (−46.7%)	15 (0.0%)	15 (0.0%)
*Q* score of papers	0.8182 ± 0.0007	0.8034 ± 0.0008 (−1.8%)	0.8254 ± 0.0023 (+0.9%)	0.8502 ± 0.0006 (+3.9%)	0.8229 ± 0.0006 (+0.6%)	0.8229 ± 0.0006 (+0.6%)
Release of scientific information (per week)	27.4 ± 0.2	28.0 ± 0.3 (+4.3%)	37.4 ± 0.6 (+36.6%)	30.2 ± 0.2 (+10.2%)	34.4 ± 0.3 (+25.7%)	34.5 ± 0.3 (+26.0%)

Data are mean ± SD unless indicated from 100 simulation runs. HD, Hellinger distance

Parentheses indicate the relative difference for each outcome to the conventional system

For median weeks between first and last submission and final decision, SD = 0

### Reviewer effort (Table 2)

The best-performing systems were the cascade and portable peer-review systems. They had the highest deviation from the conventional system performance. The systems took about 60% less time for review of all submissions. The re-review opt-out system was also beneficial in terms of total time devoted to peer review, which was 20.5% less than in the conventional system. The immediate publication and crowdsourcing peer-review systems performed slightly worse than the conventional system.

### Scientific dissemination (Table 2)

The most beneficial systems were the cascade and portable peer-review systems. They both shortened the time to publication by about 47% and increased the average weekly release of scientific information by 36.6 and 10.2%, respectively. The average *Q* score for all articles was also higher, by 0.9 and 3.9%. Moreover, the portable system published 7.0% papers more than the conventional system, but the cascade system 5.3% less. The re-review opt-out system was also beneficial in terms of papers published per year (7.4% higher), median time to publication (6.7% less) and average weekly release of scientific information (2.6% higher). Finally, the crowdsourcing and immediate publication systems differed from the conventional only in terms of release of scientific information, which was 26.0% higher for both systems.

### Overall evaluation of the systems

We considered that a system could be more efficient than the conventional system only if it improved all types of outcome measures and beneficial if it improved at least one outcome without deteriorating peer-review efficiency. Among all alternatives, only the cascade system was more efficient than the conventional system. Moreover, the crowdsourcing and immediate-publication systems were beneficial in terms of scientific dissemination. The re-review opt-out, while advantageous in some of the measures, severely deteriorated peer-review efficiency. Finally, the portable peer review was advantageous in terms of almost all outcome measures but failed to match at least the performance of the conventional system in terms of separation of *Q* score distributions.

### Sensitivity analyses

#### Exploration of the parameter space for the cascade peer-review system (Table 3)

Most of the different configurations of the cascade system surpassed or matched the performance of the conventional system in peer-review efficiency (apart from *P*_cas_ ≤ 0.80) and reviewer effort measures and all outperformed the system in median time to the final decision and release of scientific information. However, the number of published papers was lower for all alternative systems than the conventional system. The best-performing configuration was the one with *α* = 1.0, whereby the editors never asked for new reviews on resubmitted papers.

**Table 3:** Values for all outcome measures for all configurations of the cascade system

Outcome measures	$N_{g} =$			$\alpha =$		$P_{\text{cas}} =$		
	2	3	5	0.0	1.0	0.9	0.8	0.7
*Peer*-*review efficiency*								
Separation of the *Q* score distribution (HD)	0.444 ± 0.005 (−1.7%)	0.449 ± 0.008 (−0.6%)	0.458 ± 0.004 (+1.4%)	0.451 ± 0.007 (+0.2%)	0.456 ± 0.008 (+0.9%)	0.429 ± 0.004 (−5.1%)	0.411 ± 0.004 (−9.0%)	0.38 ± 0.05 (−15.9%)
Relative improvement of *Q* score	6.41 ± 0.02% (+2.2%)	6.35 ± 0.03% (+1.3%)	6.21 ± 0.04% (−1.0%)	5.95 ± 0.04% (−5.2%)	6.24 ± 0.04% (−0.4%)	6.09 ± 0.04% (−2.8%)	5.90 ± 0.03% (−5.8%)	5.73 ± 0.03% (−8.6%)
*Reviewer effort*								
Time spent in peer review (work years/year)	359 ± 8 (+0.2%)	358 ± 5 (+0.4%)	358 ± 6 (+0.4%)	540 ± 10 (+50.1%)	211 ± 5 (−41.0%)	424 ± 5 (+17.9%)	491 ± 7 (+36.6%)	557 ± 7 (+54.9%)
*Scientific dissemination*								
Annual no. of publications	27,422 ± 195 (−7.9%)	28,724 ± 368 (−3.5%)	30,182 ± 320 (+1.4%)	29,286 ± 258 (−1.6%)	29,794 ± 455 (+0.1%)	29,216 ± 354 (−1.8%)	28,409 ± 187 (−4.5%)	27,602 ± 150 (−7.2%)
Time between first submission and final decision (weeks), median	7 (−11.5%)	7 (−11.5%)	8 (+1.2%)	8 (+1.2%)	7 (−11.5%)	8 (+1.2%)	8 (+1.2%)	8 (+1.2%)
*Q* score of papers	0.810 ± 0.001 (−1.9%)	0.820 ± 0.003 (−0.7%)	0.8272 ± 0.0018 (+0.2%)	0.8197 ± 0.0011 (−0.7%)	0.8268 ± 0.0023 (+0.2%)	0.8010 ± 0.0011 (−2.9%)	0.8102 ± 0.0011 (−1.8%)	0.8293 ± 0.0023 (+0.5%)
Release of scientific information (per week)	41.8 ± 0.3 (+11.6%)	39.4 ± 0.7 (+5.2%)	36.2 ± 0.4 (−3.3%)	36.6 ± 0.3 (−2.2%)	37.5 ± 0.7 (+0.2%)	34.7 ± 0.5 (−7.3%)	32.4 ± 0.4 (−13.5%)	30.1 ± 0.3 (−19.6%)

Data are mean ± SD unless indicated from 100 simulation runs. HD, Hellinger distance

Parentheses indicate the relative difference for each outcome to the conventional system

For median weeks between first and last submission and final decision, SD = 0

#### Effect of the editor’s decision and online comments on the crowdsourcing system (Table 4)

All the different configurations of the crowdsourcing system matched or over-performed the conventional system in terms of peer-review efficiency and weekly release of scientific information but without providing any advantage in reviewer effort and the other scientific dissemination measures.

**Table 4:** Values for all the outcome measures for all different configurations of the crowdsourcing system

Outcome measures	Crowd-sourcing	Immediate publication	1 online comment	5 online comments	20 online comments	Average of all comments and reviews	Randomly select one comment or review
*Peer*-*review efficiency*							
Separation of the *Q* score distribution (HD)	0.448 ± 0.003	0.447 ± 0.003 (−0.2%)	0.446 ± 0.002 (−0.4%)	0.448 ± 0.002 (0%)	0.449 ± 0.003 (+0.2%)	0.468 ± 0.002 (+4.5%)	0.434 ± 0.001 (−3.1%)
Relative improvement of *Q* score	5.66 ± 0.05%	5.65 ± 0.03% (−0.02%)	5.67 ± 0.05% (+0.2%)	5.65 ± 0.05% (+0.3%)	5.67 ± 0.05% (+0.2%)	5.82 ± 0.01% (+2.9%)	5.51 ± 0.03% (−2.6%)
*Reviewer effort*							
Time spent in peer review (work years/year)	991 ± 12	995 ± 14 (+0.4%)	990 ± 12 (−0.2%)	989 ± 17 (−0.3%)	1000 ± 9 (+0.8%)	1009 ± 6 (+1.7%)	986 ± 7 (−0.6%)
*Scientific dissemination*							
Annual no. of publications	31,143 ± 158	31,199 ± 178 (+0.2%)	31,161 ± 152 (+0.1%)	31,052 ± 114 (−0.3%)	31,129 ± 156 (0.0%)	30,682 ± 188 (−1.5%)	31,299 ± 157 (+0.5%)
Time between first submission and final decision (weeks), median	15	15 (0.0%)	15 (0.0%)	15 (0.0%)	15 (0.0%)	15 (0.0%)	15 (0.0%)
*Q* score of papers	0.8229 ± 0.0006	0.8229 ± 0.0006 (0.0%)	0.8229 ± 0.0005 (0.0%)	0.8226 ± 0.0004 (0.0%)	0.8225 ± 0.0005 (0.0%)	0.8231 ± 0.0004 (0.0%)	0.8225 ± 0.0007 (0.0%)
Release of scientific information (per week)	34.4 ± 0.3	34.5 ± 0.3 (+0.3%)	34.5 ± 0.4 (+0.3%)	34.4 ± 0.2 (0.0%)	34.5 ± 0.2 (+0.3%)	34.2 ± 0.2 (−0.6%)	34.5 ± 0.3 (+0.3%)

Data are mean ± SD unless indicated from 100 simulation runs. HD, Hellinger distance

Parentheses indicate the relative difference for each outcome to the conventional system

For median weeks between first and last submission and final decision, SD = 0

## Discussion

We implemented several structural modifications to an original ABM of the conventional scientific publication and peer-review system and modelled five alternative peer review systems to compare their performance and relative efficiency in terms of certain outcomes. In our simulations, cascade peer review was the only alternative more efficient than the conventional one. Cascade peer review is based on the trade-off between agreeing to submit and publish in journals of lower reputation and publishing faster than usual. Under our assumptions, the number of total annual publications slightly decreased by about 5.3%, but the total time reviewers devoted to peer review decreased by 62.9% and the total time from first submission to final decision decreased by 47.3%. These results came without deterioration in the peer-review efficiency measures and even with some improvement. Most notably, this system increased the average weekly release of scientific information by 36.6%, outperforming even the two systems with immediate publication.

We did not reallocate the time researchers saved from peer review to more resources available for research, and thus we might have underestimated the advantages of both cascade and portable peer-review. For example, this reallocation of resources could lead to higher-quality review reports because reviewers are not overburdened with the task. This reallocation could also help reviewers in the re-review opt-out system raise their overall screening ability. Moreover, this time not spent on peer review could also be reallocated to more resources for research and thus raise the average *Q* score of manuscripts. Still, the systems with immediate publication release fast new information, which is reallocated to the authors as a small bonus in scientific level. However, the fact that research can be communicated very fast is something that in reality can benefit the world way more than our simulations can portray.

From the similarities and differences between the results of the two review-sharing systems, we can see how their microscopic assumptions affect the macroscopic picture. First, only their review-sharing aspect led to results of the time metrics decreasing in comparison to the conventional system. Cascading submissions to journals of lower impact factor did not affect the speed of publication and did not provide any personal advantages to authors. This occurred in cascade peer review because any paper of low *Q* score submitted in a journal network that did not include journals of very low standards would most of the time be rejected. However, cascading submissions provided some overall advantages by better separating the *Q*-score distributions because of the rejection of papers that would have been published in the portable system.

On further investigation of the configurations of the cascade system, its main configuration was not the only one providing these advantages. The best-performing configuration was the one in which the editors never asked for new reviews for any resubmitted paper. This configuration required 41% less time reviewers devoted to peer review than with the main configuration and one week less time to a final decision. This result is important because if papers were evaluated only once, they would require about 78% less time from reviewers than what they do now. However, in real life this rule could be potentially abused by reviewers with, for example, competitive motives resulting in manuscripts with unfair reviews carried forward along resubmissions. The passing of reviews should therefore not be implemented strictly and editors should always be able to ask for additional reviews if reviews appear overly negative. Moreover, we explored how the number of the journal groups affected the results. This kind of exploration essentially affected the gap in impact factor for journals between resubmissions of rejected manuscripts. The differences in number of groups of journals did not affect the results greatly, with the exception that for two or three groups, it took one week less to a final decision. To be more efficient than the conventional, the cascade system requires that the authors accept more than 90% of the time the editor’s proposal to send their paper to a journal of lower impact factor along with the reviews.

The system of portable peer review was modelled exactly as the cascade system, with the only exception that authors were not restricted by journal groups when resubmitting. Our results suggest that this system is also beneficial, almost as much as the cascade system. However, the 4.2% decrease in separation between the *Q* score distributions of the published and unpublished papers is undesirable. The portable system, despite its small disadvantage in separation of *Q* score distributions, might be easier to implement in real-life because it provides authors with more freedom to resubmit.

The system of crowdsourcing online reviews was beneficial but not more efficient than the conventional system. Simply by implementing its immediate publication version, without online reviews, increased the release of scientific information by 25.7%. Then, introducing online reviews to the system increased both peer-review efficiency outcome measures because of more correct evaluation of papers due to the fact that editors obtain more reviews. Online reviews are rarely as detailed as those from invited reviewers and thus we assumed that the editor assigned them lower weight than the invited ones. Moreover, since the results for only one online review per paper are the same as those for 5 or 20, averaging all the online reviews did not affect our outputs. Finally, in the extreme case, in which all online reviews were as detailed as the invited ones and all would be equally averaged, the system clearly managed to separate the *Q* score distributions better than any other. However, when we randomly selected one review, the system matched the behaviour of the conventional system.

The system of re-review opt-out is conceptually easy to implement however failed to at least match the performance of the conventional system on the two peer-review efficiency measures but improved on almost all the remaining outcome measures. In our implementation, we substituted the second round of revisions by the reviewers with an editorial evaluation. Thus, a real-life experiment and extra modelling efforts are needed to validate whether we obtained these results for the two outcome measures due to our modelling assumptions, which give high importance to the second peer-review round, or because this system is really less efficient than the conventional one.

A limitation of our simulations is that to our best knowledge no data currently exists for any of the five implemented alternative peer-review systems. For this reason, we had to obtain results by comparing the alternatives with the conventional system. However, these alternatives are not yet fully implemented and much of the relevant data are not even generated to date. A second limitation is that our results are likely affected by our assumptions and choices, more than the general idea behind these alternative systems. In general, we tried to adopt the most reasonable implementations of these alternatives in our main simulations and to test their limits and our assumptions by further exploring the parameter space for the two most important systems. Finally, our outcome measures were based on variables that are abstract in how we measured them. In theory, papers have a *Q* score that can act as a proxy of their novelty and correctness, for instance, and information is disseminated when journals publish new papers. However, because we lack a universally agreed-upon method and variables that measures these values, we needed to create them to help inform our decisions. These assumptions can only be proven or disproven after real-life experiments.

## Conclusions

We compared the efficiency of five alternative peer-review systems to the conventional system by using an ABM approach. Only the cascade system was more efficient than the conventional system in all three types of outcomes. The portable system closely matched the cascade system’s performance and was more efficient than the conventional system in all but one measure. Moreover, all the configurations of the crowdsourcing system were beneficial and managed to match or improve the peer-review efficiency and scientific information measures but without any important change in the other measures. Finally, we recommend prioritizing a system of review sharing to create a sustainable scientific publication and peer-review system.
